# Echinacoside alleviates Ang II-induced cardiac fibrosis by enhancing the SIRT1/IL-11 pathway

**DOI:** 10.22038/ijbms.2024.79837.17296

**Published:** 2025

**Authors:** Yingbiao Wu, Zhongping Ning

**Affiliations:** 1 Department of Cardiology, Shanghai Pudong New Area Zhoupu Hospital (Shanghai Health Medical College Affiliated Zhoupu Hospital Shanghai; 2 201318, China

**Keywords:** Angiotensin IIm Cardiac fibrosis Echinacoside, Heart failure, Sirtuin1

## Abstract

**Objective(s):**

Echinacoside (ECH) is an anti-fibrotic phenylethanoid glycoside derived from the *Cistanche* plant that protects against cardiac dysfunction by mitigating apoptosis, oxidative stress, and fibrosis. Nevertheless, ECH’s precise function and mechanisms in addressing cardiac fibrosis are still not fully understood.

**Materials and Methods:**

In our current investigation, we induced cardiac fibrosis in mice by administering Angiotensin II (Ang II) and subsequently assessed the effects of ECH treatment four weeks post-fibrosis induction. Additionally, in an *in vitro* setting, we exposed cardiac fibroblasts (CFs) to Ang II to prove the anti-fibrotic mechanisms of ECH.

**Results:**

ECH treatment effectively reversed cardiac fibrosis in the mice model. ECH treatment significantly reduced the levels of fibrosis-related genes, such as α-SMA, Collagen I, and Collagen III (all, *P<*0.001). Moreover, it reduced the number of apoptotic cells and regulated the expression of apoptosis-related genes, such as BAX and BCL-2 (all, *P<*0.001). ECH treatment also positively affected serum levels of markers associated with cardiac fibrosis, including LDH, CK-MB, ANP, BNP, CTnl, and CTnT (all, *P<*0.001), in the *in vivo* experiments. In the *in vitro* studies, ECH pretreatment alleviated cardiac fibroblast apoptosis and reduced cell migration, collagen deposition, and MMP expression (all, *P<*0.001). In our *in vivo* and *in vitro *investigations, we observed that ECH treatment reversed the down-regulation of SIRT1 and up-regulation of IL-11 following cardiac fibrosis. The results suggest that the protective effects of ECH may involve regulating the SIRT1/IL-11 pathway.

**Conclusion:**

ECH may protect against Ang II-induced cardiac fibrosis via the SIRT1/IL-11 pathway.

## Introduction

Cardiac fibrosis (CF) represents a significant global health concern and is closely associated with various cardiovascular conditions, including myocardial infarction, hypertensive heart disease, and cardiomyopathy (1). In CF, pathological insults within the myocardium trigger the activation of resident cardiac fibroblasts into myofibroblasts, leading to myocardial damage, increased secretion and deposition of the extracellular matrix (primarily type I and III collagen), and expression of α -smooth muscle actin (α-SMA), which contributes to matrix stiffness and contractile function (2,3). Myofibroblasts possess anti-inflammatory properties and produce cytokines, extracellular matrix components, and paracrine factors that aid wound healing (4). Within the cardiac interstitium, secreted cytokines and growth factors, such as Tumor Necrosis Factor (TNF-α), Interleukin (IL)-1, IL-6, IL-10, IL-11, chemokines, and members of the Transforming Growth Factor-β family, play diverse roles in the fibrotic response (5-7). Regrettably, no effective pharmaceutical treatment for CF is currently available. Consequently, it is imperative to develop effective therapies, explore the underlying cell biological mechanisms, study the functional implications of fibrotic changes in the myocardium, and identify potential therapeutic targets to effectively combat fibrosis and delay the onset of heart failure (HF).

Angiotensin II (Ang II) is a pivotal mediator of CF, acting through two receptor types, angiotensin type I and II. Ang II is recognized for its role in promoting fibrosis, stimulating fibroblast proliferation, enhancing the secretion and deposition of extracellular matrix proteins, and contributing to cardiac remodeling (8-10).

Echinacoside (ECH) is a naturally produced phenylethanoid glycoside with numerous pharmacological activities, such as anti-oxidant, anti-inflammatory, anti-neurotoxic, anti-apoptotic, and anti-tumor properties (11-15). Current study reports have highlighted its cardioprotective effects, with ECH contributing to cardiac remodeling and improved heart function by inhibiting mitochondrial reactive oxygen species (ROS) (16). ECH also suppresses cardiomyocyte pyroptosis, a critical factor in cardiac remodeling and heart function (17). Nevertheless, ECH’s specific role and underlying mechanisms in countering Ang II-induced CF remain to be elucidated.

Sirtuin1 (SIRT1) is well-documented for its therapeutic potential and protective role in cardiovascular diseases. Numerous studies have demonstrated SIRT1’s significant impact on fibrosis in various organs, including the heart, kidney, and liver (18, 19). SIRT1 functions to reduce CF by impeding oxidative stress (20), making it a promising target for novel treatment strategies aimed at preventing CF (21). Despite these promising findings, the precise mechanism of SIRT1 activation remains unclear. Notably, the question of whether ECH can effectively inhibit Ang II-induced CF through the activation of SIRT1 still needs to be answered, both *in vivo* and *in vitro*.

Interleukin-11 (IL-11), a multifunctional cytokine within the IL-6 family, presents a complex role in disease progression. Alongside IL-6, IL-27, IL-31, LIF, OSM, CNTF, CT-1, cardiotrophin-2 (CT-2), and cardiotrophin-like cytokine (CLC)(22-24), IL-11 plays a critical role in initiating and advancing various diseases by modulating inflammatory responses in the specific inflammatory microenvironment (25-27). Recent studies have revealed IL-11’s complexity in cardiovascular diseases, particularly in developing CF and the chronic activation of the IL-11 signaling pathway associated with heart failure (28-29). The protective function of IL-11 in acute myocardial infarction (AMI) and ischemia-reperfusion injury (IRI) has been investigated in mouse models (30, 31), adding to the intellectual challenge of understanding its involvement in Ang II-induced cardiac fibrosis.

However, this research aims to explore the role of ECH in alleviating cardiac remodeling and improving heart function. We induce a murine CF model using intraperitoneal Ang II infusion and subsequently treat CF with ECH. Our research explores the potential effects of ECH on Ang II-infused murine CF and its implications for the SIRT1/IL-11 pathway.

## Materials and Methods

***Animals***

The current investigation utilized male C57BL/6 mice (8-10 weeks old, weighing 20-25 g) as wild-type counterparts. The mice were housed in a specific pathogen-free (SPF) facility under controlled environmental conditions, with the ambient temperature set at 22±2 ^°^C and humidity maintained at 40%. They were subjected to a 12-hour light/dark cycle and provided *ad libitum* access to food and water. The animal care procedures and surgical interventions were conducted with utmost adherence to the standards outlined in the Guide for the Care and Use of Laboratory Animals, as approved by the Animal Care and Use Committees of Shanghai Pudong Zhoupu Hospital, and complied with the established guidelines for animal welfare (32).

***Animal model***

The induction of the Murine Cardiac Fibrosis model involved the subcutaneous infusion of either saline or angiotensin II (Ang II, 2.0 mg/kg/day, HY-13948, MedChemExpress) via Alzet osmotic mini-pumps (Model 2004, USA) over 28 days (33). Echinacoside (ECH, 50 mg/kg/day, HY-N0020, MedChemExpress) or an equivalent volume of saline was administered intraperitoneally once daily for the same duration. A total of 24 mice were randomly allocated into three groups, each comprising eight mice. The groups were as follows: (1) Control group: mice received saline infusion via mini-pumps and intraperitoneal saline injections once daily; (2) Ang II group: mice received Ang II infusion via mini-pumps and intraperitoneal saline injections once daily; (3) ECH group: mice received Ang II infusion via mini-pumps and intraperitoneal ECH injections once daily. 

Systolic blood pressure (SBP) and diastolic blood pressure (DBP) of the mice were assessed using a tail-cuff system (Softron BP98A; Softron Tokyo, Japan) on days 0, 7, 14, 21, and 28 post-Ang II infusion. At the end of the 28 days following Ang II infusion, all mice’s body weight (BW) was recorded, after which the mice were anesthetized, and serum samples were collected. Heart weight (HW) and tibia length (TL) were measured for each mouse to calculate the ratios of HW/BW and HW/TL. The cardiac ventricular tissues were subjected to a thorough analysis, being harvested and divided into two portions; one portion was promptly frozen in liquid nitrogen for RT-qPCR and western blot analysis, while the other part was fixed in 4% paraformaldehyde for histological examination.

***Echocardiography assessment***

Echocardiography was carried out using a Small Animal Ultrasound Imaging System (Vevo3100, VisualSonics, Canada). M-mode images were obtained, and parameters were as follows: ejection fraction (EF), fractional shortening (FS), left atrial diameter (LAD), left ventricular end systolic diameter (LVESd), and left ventricular end diastolic diameter (LVEDd)(34).

***Measurement of serum biochemical parameter***

Serum samples were collected 28 days after Ang II infusion and the myocardial enzyme lactate dehydrogenase (LDH) was analyzed (n=8 per group) using the LDH activity Assay Kit (E-BC-K046-M, Elabscience, China) according to the manufacturer’s instructions. 

***Enzyme-linked immunosorbent assay (ELISA)***

The blood samples from the mice were collected 28 days after Ang II infusion, allowed to stand for two hours, and then centrifuged at 1500 g for 15 min to obtain the serum. The serum was then divided into aliquots, transferred into cryotubes, and stored in a -80 ^°^C refrigerator. Serum protein levels of mouse ANP (E-EL-M0166c, Elabscience, China), BNP (E-EL-M0204c, Elabscience, China), cTnI (SEKM-0153, Solarbio, China), cTnT (SEKM-0150, Solarbio, China), and CK-MB (SEKM-0152, Solarbio, China), and IL-11(DY418, R&D Systems, USA) were quantified by ELISA assay with commercially available kits. The assays were performed according to the manufacturer’s instructions. 

***Histology***

The cardiac ventricular tissues of mice were subjected to various analyses to assess fibrosis and apoptotic cell populations. Masson’s trichrome staining was employed to evaluate fibrosis, and the extent of fibrosis was quantified by determining the ratio of fibrotic area to normal myocardium, also known as the collagen volume fraction.

An analysis was conducted using a TUNEL (Terminal deoxynucleotidyl transferase dUTP Nick End Labeling) assay to examine cell death within cardiac tissues. The methodology encompassed the identification of apoptotic cells by utilizing a one-step TUNEL assay kit (Beyotime Biotechnology, China) per the manufacturer’s guidelines. Subsequently, TUNEL-positive cells were enumerated and assessed with the Olympus BX5 imaging system (Olympus America, Melville, NY, USA) (35).

Immunohistochemistry staining was conducted to examine the presence of interleukin-11 (IL-11) in the cardiac ventricular tissues. The process involved quenching endogenous peroxidase with 3% H_2_O_2_ and blocking non-specific binding with 2% bovine serum albumin (Amresco, Solon, OH). The cardiac tissue sections were exposed to an IL-11 antibody (Im-02976B, YaJi Biological, China) overnight at 4 ^°^C. The samples then underwent treatment with biotinylated secondary antibodies, a crucial step in our procedure, and were thoroughly rinsed to ensure the removal of any excess or unbound antibodies. Positive staining was detected by utilizing DAB Substrate (Cwbiotech, Beijing, China) following the protocol outlined in the ABC Kit (Vector Laboratories, Burlingame, CA, USA).

***Isolation and culture of rat cardiac fibroblasts***

An intraperitoneal injection of pentobarbital sodium (60 mg/kg) was used to induce anesthesia in neonatal SD rats. Then, thoracotomy was carried out, the heart was isolated, and ventricles were cut and placed in a sterile petri dish. The heart tissues were cut into pieces of about 1 mm^3^ in size and were filtered through a filter (200-mesh). Heart fibroblasts were isolated and grown in DMEM containing 10% fetal bovine serum (FBS) at 37 ^°^C and 5% CO_2_ following trypsin digestion and centrifugation. The culture medium was changed every three days. An inverted microscope was used to visualize and capture cell morphology images. The cardiac fibroblasts were identified using Vimentin staining and photographed under a fluorescence microscope (36).

***Cell culture***

The cells were cultivated at 37 ^°^C with 5% CO_2_ in a strictly controlled, humidified environment using DMEM containing 10% FBS. After two hours of pretreatment with ECH (20 μM), cells were incubated with or without Ang II (1 μM) for 48 hr under the same conditions. In the inhibitor experiment, SIRT1 activity was inhibited using EX-527 (10 μM, HY-15452, MedChemExpress).

***Cell migration assay***

An 8-μm Transwell system (BD Biosciences, San Diego, CA, USA) was used to perform the cell migration assay. The primary fibroblasts were plated at 5×10^4^ cells per well to the top chamber with 500 μl of serum-free medium. The bottom well was filled with complete medium. Cell migration was allowed to proceed after a 24 hr incubation at 37 ^°^C in a tissue culture chamber. After removing the non-migratory cells from the upper chamber, the migratory cells were stained with 0.01% Crystal Violet (Sigma) and fixed with 4% paraformaldehyde. The cells were counted using microscopy at six random fields (magnification x 200) (37). All migration assays were repeated at least twice with similar results.

***Cellular immunofluorescence***

The cellular immunofluorescence assay was performed to detect vimentin and α-SMA in cardiac fibroblasts. Following a 20 min fixation with 4% paraformaldehyde, the cells were treated for 15 min at room temperature (RT) with 0.5% Triton X-100. Following a two-hour RT blocking step with 5% goat serum, the cells were incubated for an overnight period at 4 ^°^C with the primary antibodies Vimentin (1:400; ab92547; Rabbit monoclonal, Abcam) and α-SMA (1:500; ab7817; mouse monoclonal, Abcam, UK). The secondary antibodies applied were Alexa Fluor 488 (Ab150113, Abcam) and photographed under a fluorescence microscope (Magnification: ×400) (37).

***Intracellular ROS level evaluation***

Fibroblast cells were plated at 5×10^4^ cells/ml on glass slides (Ibidi, Martinsried, Germany) to measure the intracellular ROS level. After being incubated for 48 hr, the cells were washed with PBS and then incubated for 30 min at 37 ^°^C with DHE fluorescent dye (DHE: S0063, Beyotime). Following a wash, the cells were fixed for 15 min with PFA (4%). Under a fluorescent microscope, the cells were captured on camera (magnification x 200). After using Image J to thoroughly analyze the acquired images, quantification was done by measuring the fluorescence of 10 ROI in each image, each in triplicate.

***Measurement of oxidative stress indicators***

The assessment of the oxidative stress-related enzyme SOD, CAT, and MDA was performed following the protocols provided by each commercial kit (MDA: S0131S; SOD: S0109; and CAT: S0051; Beyotime, Shanghai, China). The measurement was obtained using spectrophotometric techniques and a plate reader from Bio-Rad Laboratories, Inc. (Hercules, California, USA). SOD, CAT, and MDA were analyzed with microplate reader 450 nm, 570 nm, and 532 nm. The results were expressed as U/ml for SOD and CAT U/ml. The protein concentration of MDA was expressed as nmol/mg (38).

***RT-qPCR***

Total RNA was isolated utilizing the Trizol method (Invitrogen) and subjected to reverse transcription to generate cDNA employing the Prime Script (R) RT reagent kit (TaKaRa Bio Inc, Dalian, China). Utilizing the SYBR Premix Ex Taq kit (TaKaRa Bio Inc), real-time PCR was conducted in triplicate on a RealTime PCR System (Applied Biosystems, Foster City, CA, USA). The primer sequences targeting Mouse (α-SMA, Collagen I, Collagen III, BAX, BCL-2, IL -11, SIRT1, and GAPDH) and Rat (α-SMA, Collagen I, Collagen III, Fibronectin, CTGF, MMP9, NOX2, NOX4, and GAPDH) are detailed in Table 1. The PCR conditions were established as denaturation at 94 ^°^C for 10 min, succeeded by 40 cycles at 94 vC for 15 sec and 58 ^°^C for 30 sec. The obtained data were standardized to GAPDH as an internal reference, and the RNA levels were evaluated using the 2^-ΔΔCt^ approach. 

***Western blotting***

The ice-cold RIPA buffer was used to lyse the cardiac tissue and cells (Pierce, Pittsburgh, PA, USA). The protein concentration was measured using the BCA protein assay kit after the lysates were centrifuged for 10 min at 10,000 g. Proteins were separated on a 10% acrylamide gel using SDS-PAGE. The PVDF membrane was transferred to the gel and then blocked with 5% nonfat milk. Following this, the primary antibodies for SIRT1 (1:400, sc-74465, mouse monoclonal, Santa Cruz, USA), IL-11 (1:500, sc-133063, mouse monoclonal, Santa Cruz, USA), Wnt (1:500, ab15251, rabbit polyclonal, Abcam), β-Catenin (1:500, ab3257, rabbit monoclonal, Abcam), and GAPDH (1:2000, ab9485, rabbit polyclonal, Abcam) were incubated at 4 ^°^C overnight. The membrane was further incubated for two hours using an HRP-conjugated secondary antibody (Kangchen Biotech, Beijing, China). The band captured by the FluorChem Image System (Alpha Innotech, Santa Clara, CA, USA) was visualized on the membrane by applying SuperSignal West Pico Chemiluminescent Substrate (Pierce, Pittsburgh, PA, USA).

***Statistical Analysis***

The data was thoroughly and comprehensively analyzed using the statistical program SPSS 20.0. Group comparisons were meticulously scrutinized using one-way ANOVA with Tukey’s test, with a significance criterion set at *P*<0.05.

**Table 1:** List of oligonucleotide primer sequences used in this research

Genes	Forward primer (5′-3′)	Reverse primer (5′-3′)
Mouse α-SMA	TCCTGACGCTGAAGTATCCGATA	GGCCACACGAAGCTCGTTAT
Mouse Collagen I	GCTCCTCTTAGGGGCCACT	CCACGTCTCACCATTGGGG
Mouse Collagen III	TCCCCTGGAATCTGTGAATC	TGAGTCGAATTGGGGAGAAT
Mouse BAX	GCCTCCTCTCCTACTTCGG	AAAAATGCCTTTCCCCTTC
Mouse BCL-2	CTCGTCGCTACCGTCGTGACTTCG	CAGATGCCGGTTCAGGTACTCAGTC
Mouse IL-11	CTGCCCACCTTGGCCATGAG	CCAGGCGAGACATCAAGAAAGA
Mouse SIRT1	TCGGCTACCGAGGTCCATA	AACAATCTGCCACAGCGTCA
Rat Collagen I	GCCTCAGCCACCTCAAGAGA	GGCTGCGGATGTTCTCAATC
Rat Collagen III	CCAGGACAAAGAGGGGAACC	CCATTTCACCTTTCCCACCA
Rat α-SMA	CTATTCCTTCGTGACTACT	ATGCTGTTATAGGTGGTT
Rat Fibronectin	GGATCCCCTCCCAGAGAAGT	GGGTGTGGAAGGGTAACCAG
Rat CTGF	GTGTGCACTGCCAAAGATG	TCGGTAGGCAGCTAGGGC
Rat MMP-9	CCCTGCGTATTTCCATTCATC	ACCCCACTTCTTGTCAGCGTC
Rat NOX2	CTGCCAGTGTGTCGGAATCT	TGTGAATGGCCGTGTGAAGT
Rat NOX4	GAACCCAAGTTCCAAGCTCA	GCACAAAGGTCCAGAAATCC
Mouse GAPDH	ACTCCACTCACGGCAAATTC	TCTCCATGGTGGTGAAGACA
Rat GAPDH	CCCCCAATGTATCCGTTGTG	TAGCCCAGGATGCCCTTTAGT

**Figure 1:**
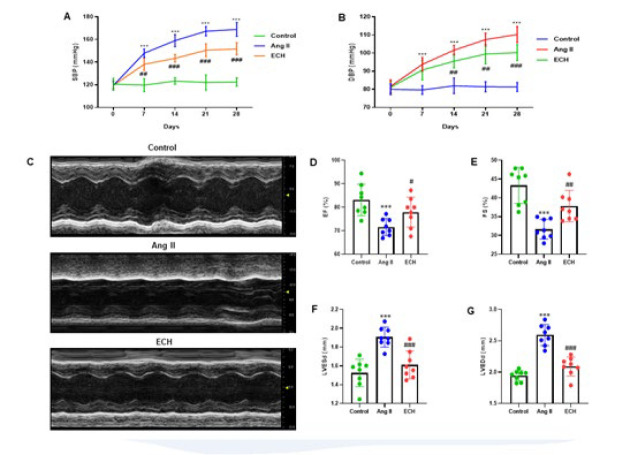
Echinacoside improves cardiac dysfunction of Ang II-infused mice

Mice were injected intraperitoneally with echinacoside (ECH, 50 mg/kg/day), followed by subcutaneous infusion of Ang II (2.0 mg/kg/day) for 28 d. (A, B) The mice’s blood pressure (SBP and DBP) was measured at 0, 7, 14, 21, and 28 days after the start of Ang II infusion. ECH reduced SBP and DBP. (C) Representative images of M-mode echocardiography. ECH markedly improved the echocardiographic parameters, increased (D) EF and (E) FS, and decreased (F) LVESd and (G) LVEDd. Data are presented as mean±SD (n=8 in each group) with at least three independent experiments. One-way ANOVA with Tuckey’s test was used for analysis. ***P<0.001 vs Control group; ^##^*P<*0.01, ^###^*P<*0.001 vs Ang II group

Ang II, angiotensin II; ECH, echinacoside; SBP, systolic blood pressure; DBP, diastolic blood pressure; EF, ejection fraction; FS, fractional shortening; LVESd, left ventricular end-systolic diameter; LVEDd, left ventricular end-diastolic diameter

**Figure 2:**
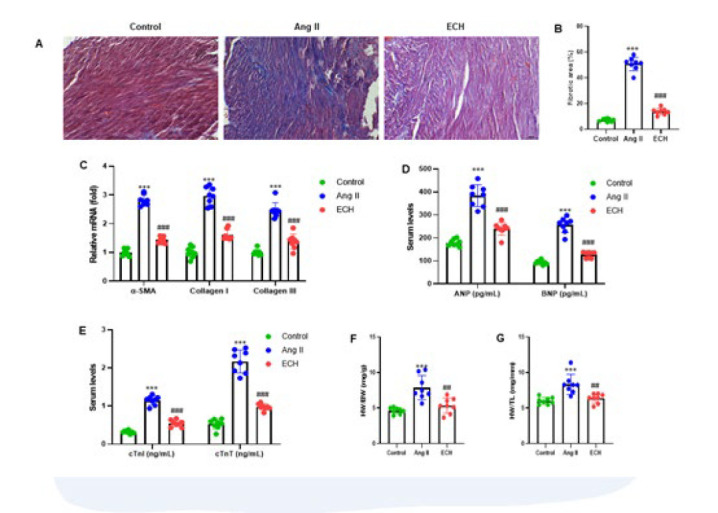
Echinacoside suppresses cardiac fibrosis and reduces heart size in Ang II-infused mice

(A) Left ventricular muscle tissues were stained with Masson trichrome to evaluate cardiac fibrosis, and the representative images are shown (Magnification 200×). (B) Quantification of cardiac fibrosis by calculating the percentage of the fibrotic area. (C) RT-qPCR was applied to determine the mRNA expression α-SMA, Collagen I (COL1A1), and Collagen III (COL3A1). ELISA was applied to measure the serum levels of (D) ANP, BNP, and (E) cTnI, cTnT in mice. (F, G) Heart size of mice was evaluated by HW/BW and HW/TL ratios. Data are presented as mean±SD (n=8 in each group). ***P<0.001 vs control group; ^###^*P<*0.001 vs Ang II group

α-SMA, α-smooth muscle actin; ANP, atrial natriuretic peptide; BNP, brain natriuretic peptide; cTnI, cardiac troponin I; cTnT, cardiac troponin T

**Figure 3:**
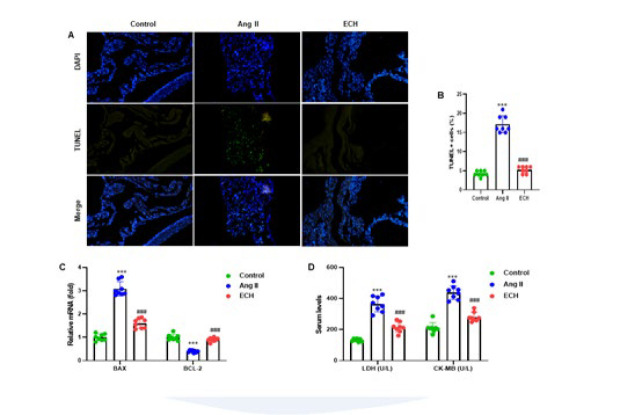
Echinacoside reduces apoptosis of cardiac tissue in Ang II-infused mice

(A) Left ventricular muscle tissue was stained with TUNEL and DAPI (200×). (B) Quantification of the percentage of TUNEL-positive cells relative to DAPI-stained cells. (C) RT-qPCR was applied to determine the mRNA expression of two apoptosis genes, BAX and BCL-2. GAPDH as an internal control. (D) Serum levels of LDH and CK-MB were measured by colorimetry and ELISA, respectively. Data are presented as mean±SD (n=8 in each group). ***P<0.001 vs control group; ^###^*P<*0.001 vs Ang II group

LDH, lactate dehydrogenase; CK-MB, creatine kinase isoenzyme MB

**Figure 4:**
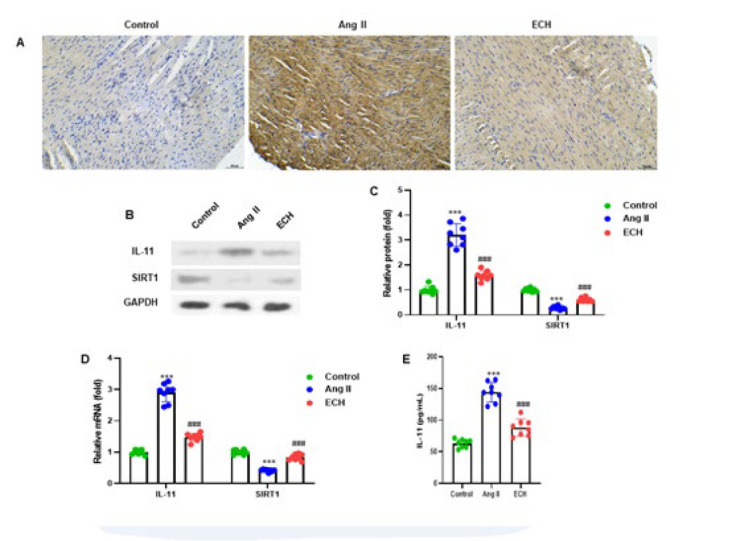
Echinacoside regulated SIRT1-IL-11 pathway in cardiac tissue of Ang II-infused mice

(A) Representative images of immunohistochemistry in left ventricular muscle tissue by IL-11 (200×). (B) Western blot was applied to determine the protein expression of IL-11 and SIRT1, and their representative bands are shown. (C) Quantification of protein bands of IL-11 and SIRT1. Protein expression was normalized to GADPH. (D) RT-qPCR was applied to determine the mRNA expression of IL-11 and SIRT1. (E) Serum IL-11 concentrations were measured by ELISA. Data are presented as mean±SD (n=8 in each group). ***P<0.001 vs control group; ^###^*P<*0.001 vs Ang II group

**Figure 5:**
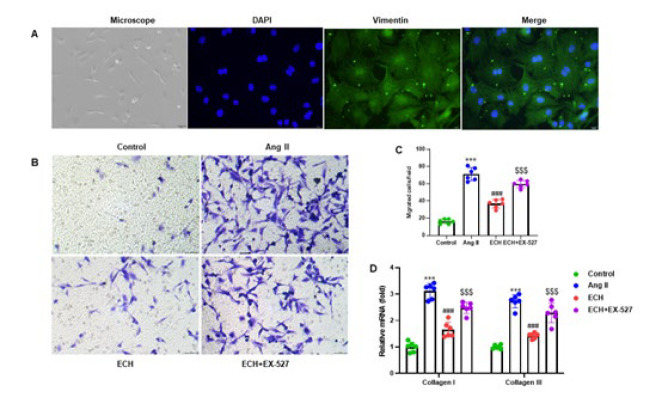
Echinacoside inhibits Ang II-induced migration of rat cardiac fibroblasts

(A) Primary cardiac fibroblasts were isolated from the ventricular myocytes of neonate SD rats and identified by Vimentin staining. The representative images of cardiac fibroblasts photographed under an inverted microscope (200×) or immunofluorescence are shown (400×). (B) Cardiac fibroblasts were pretreated with a SIRT1 inhibitor EX-527 (10 μM) and ECH (20 μM) for 2 hr, followed by incubation with Ang II (1 μM) for a further 48 hr. Cell migration was assessed by Transwell assay. (C) The average number of migrated cells per field from 6 random fields. (D) RT-qPCR was applied to determine the mRNA expression of Collagen I (COL1A1) and Collagen III (COL3A1). GADPH served as an internal control. Data are shown as mean±SD (n=6 per group) from three independent experiments. ***P<0.001 vs the control group; ^###^*P<*0.001 vs Ang II group; ^$$$^ *P<*0.001 vs ECH group

**Figure 6:**
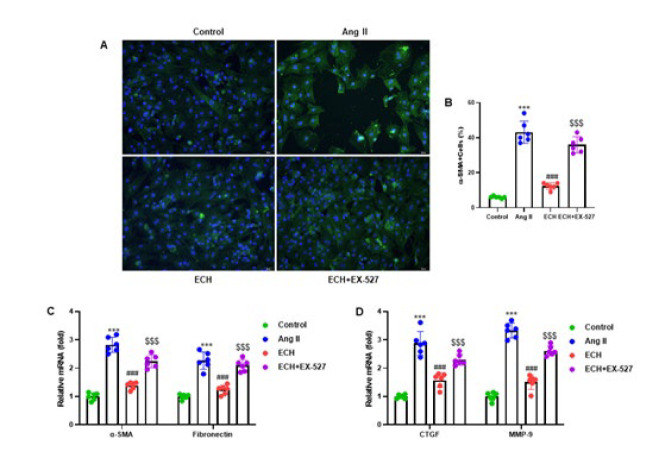
Echinacoside suppresses the Ang II-induced cardiac fibroblast differentiation

(A) Cardiac fibroblasts were stained with α-SMA antibody to evaluate their differentiation. The representative images are shown (200×). (B) Quantification of α-SMA positive cells relative to DAPI-stained cells. RT-qPCR was applied to measure the mRNA expression of (C) α-SMA (ACTA2), fibronectin, and (D) CTGF, MMP-9. GADPH served as an internal control. Data are shown as mean±SD (n=6 per group). CTGF, connective tissue growth factor; MMP-9, matrix metalloproteinases-9. ***P<0.001 vs control group; ^###^*P<*0.001 vs Ang II group; ^$$$^ *P<*0.001 vs ECH group

**Figure 7:**
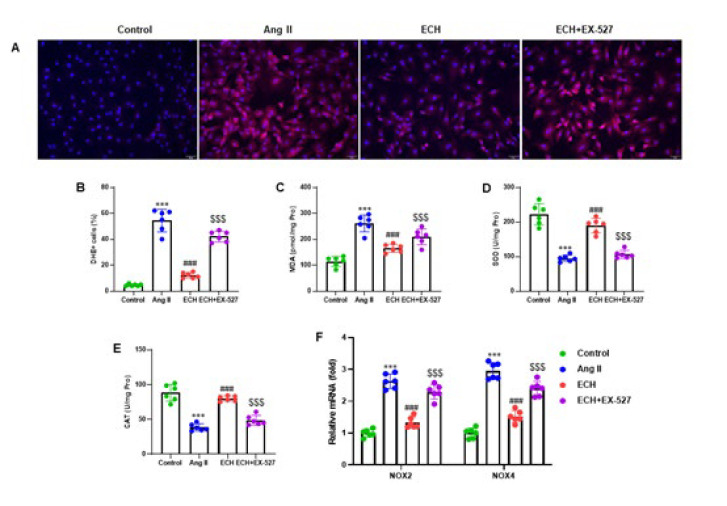
Echinacoside inhibits Ang II-induced intracellular ROS generation and oxidative stress

(A) Cardiac fibroblasts were stained with DHE to assess the intracellular ROS level. The representative image is shown (200×). (B) Quantification of intracellular ROS by calculating the percentage of DHE-positive cells. The oxidative stress indicators were detected in the cell lysate of cardiac fibroblasts: (C) MDA, (D) SOD, and (E) CAT. (F) RT-qPCR was applied to measure the mRNA expression of NOX2 and NOX4. Data are shown as mean ± SD (n=6 per group). DHE, dihydroethidium. ***P<0.001 vs control group; ^###^*P<*0.001 vs Ang II group, ^$$$^ *P<*0.001 vs ECH group

**Figure 8:**
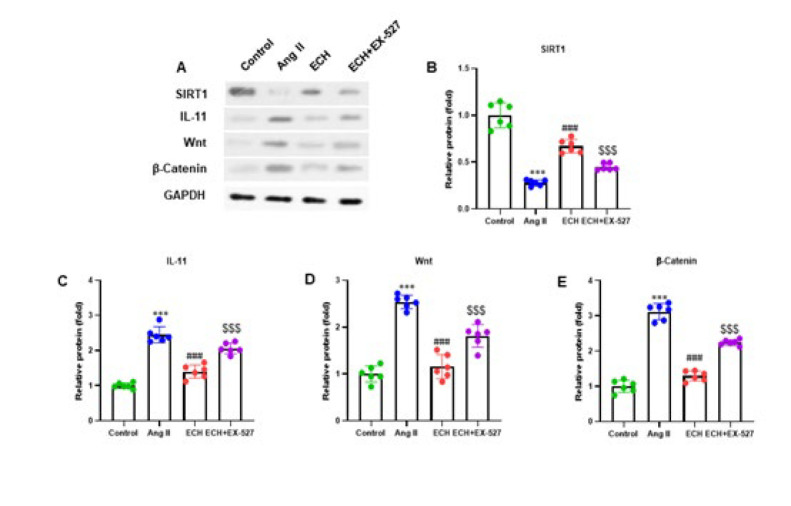
Echinacoside enhances SIRT1 and regulates the Wnt/β-Catenin and IL-11 pathway in Ang II-induced cardiac fibroblasts

(A) Representative protein bands of western blot on SIRT1, IL-11, Wnt, and β-Catenin. (B, C, D, E) Quantitative analysis of protein bands that are normalized to GAPDH. Data are shown as mean±SD (n=6 per group). ***P<0.001 vs control group; ^###^*P<*0.001 vs Ang II group, ^$$$^ *P<*0.001 vs ECH group

## Results

***Echinacoside (ECH) improves cardiac dysfunction of Ang II-infused mice***

Our study delved into the effects of ECH intervention on the hemodynamic metrics of mice subjected to Angiotensin II (Ang II) infusion. Evaluation of blood pressure, encompassing both systolic blood pressure (SBP) and diastolic blood pressure (DBP), was conducted on days 0, 7, 14, 21, and 28 subsequent to the commencement of Ang II infusion through the utilization of the tail-cuff technique. Hemodynamic data analysis revealed a consistent and significant increase in both SBP and DBP in the Ang II-treated groups than the control groups (*P*<0.01). In contrast, the groups that received ECH treatment showed a steady decrease in SBP and DBP compared to the Ang II-treated groups (*P*<0.01)(Figure 1 A, B). Furthermore, echocardiography was performed after the 28-day ECH treatment, with M-mode echocardiography images in Figure 1C. The echocardiography results clearly showed that the cardiac function, specifically ejection fraction (EF) and fractional shortening (FS), in the Ang II group was significantly impaired compared to the control groups (*P*<0.01). Conversely, ECH treatment significantly improved cardiac function, as shown in Figures 1D and E. Additionally, the left ventricular end-systolic diameter (LVESd) and left ventricular end-diastolic diameter (LVEDd) were notably increased in the Ang II-induced groups when compared to the control groups (*P*<0.001). ECH treatment, however, led to a significant reduction in LVESd and LVEDd compared to the Ang II-induced groups (*P*<0.01). Collectively, our results demonstrate that ECH effectively ameliorates cardiac dysfunction induced by Ang II.

***Echinacoside suppresses cardiac fibrosis and reduces heart size in Ang II-infused mice***

To assess CF, we employed Masson’s trichrome staining on the left ventricular muscle tissue of the mice, and a representative image is presented in Figure 2A. The degree of fibrosis was assessed by computing the proportion of fibrotic tissue to healthy myocardial tissue, known as the collagen volume fraction. Comparing the Ang II group to the control group, a substantial increase in myocardial collagen deposition was observed (*P*<0.001). In contrast, the ECH treatment group displayed a significant reduction in collagen deposition compared to the Ang II group (*P*<0.001) (Figure 2B). Furthermore, we utilized RT-qPCR to analyze the mRNA expression of fibrosis-related genes (α-SMA, COL1A1, and COL3A1) in the cardiac ventricular tissues of the mice. Ang II significantly increased the expression of these three fibrotic markers, but their expression was notably attenuated by ECH treatment (*P*<0.001) (Figure 2C). In addition, we conducted ELISA assays to assess serum protein levels. The Ang II-infused mice group revealed a significant up-regulation in the expression of ANP, BNP, cTnl, and cTnT proteins in the serum compared to the control groups (all *P*<0.001). Conversely, the ECH treatment groups displayed a marked reduction in the expression of these proteins in comparison to the Ang II-infused groups (*P*<0.001)(Figure 2D, E). To evaluate changes in heart size, we performed calculations to determine the ratios of heart weight to body weight (HW/BW) and heart weight to tibia length (HW/TL). The ECH treatment groups exhibited a significant reduction in heart size compared to the Ang II-infused groups (*P*<0.01)(Figure 2F, G). Our findings demonstrate that ECH significantly attenuated CF and heart size induced by Ang II.

***Echinacoside reduces apoptosis of cardiac tissue in Ang II-infused mice***

We employed TUNEL staining to investigate the impact of ECH on apoptosis in ventricular tissue. The tissues were subjected to TUNEL assay and subsequently counterstained with DAPI. Our results clearly indicated a reduction in apoptosis in mice treated with ECH following Ang II exposure, as evidenced by the diminished green fluorescence in the mouse tissue (Figure 3A). Quantitative analysis further supported these findings, revealing a significant reduction in the number of TUNEL-positive cells in ECH-treated mice compared to those subjected to Ang II (*P*<0.001) (Figure 3B). Additionally, our RT-qPCR analysis demonstrated significant alterations in the mRNA levels of two apoptosis-associated genes, BAX and BCL-2, in mice exposed to Ang II and in those receiving ECH treatment (*P*<0.001) (Figure 3C). Moreover, through colorimetry and ELISA, we observed that ECH effectively attenuated the increased serum levels of LDH and CK-MB induced by Ang II in mice (*P*<0.001)(Figure 3D). The results collectively suggest that ECH plays a crucial role in mitigating apoptosis and reducing cardiac damage infused by Ang II.

***Echinacoside regulated SIRT1/IL-11 pathway in cardiac tissue of Ang II-infused mice***

In our study, we conducted a series of analyses on Ang II-induced cardiac left ventricular muscle tissue to investigate the role of IL-11. Figure 4A displays the representative images obtained through immunohistochemistry. We also carried out western blot analyses to measure the protein levels of SIRT1 and IL-11 in the Ang II-induced cardiac tissue, with the representative protein bands depicted in Figure 4B.

The quantification of protein levels revealed noteworthy findings. In the Ang II-induced mice group, we observed a significantly reduced SIRT1 expression, with levels decreased to 0.25-fold compared to the control group. Conversely, IL-11 expression increased substantially to 3.50-fold in the Ang II-induced group compared to the controls. However, in the ECH-treated group, there was a 0.75-fold alleviation in SIRT1 expression and a 1.25-fold reduction in IL-11 expression when compared to the Ang II-induced group (*P*<0.001) (Figure 4C).

Furthermore, we employed RT-qPCR to analyze the mRNA expression of the SIRT1 and IL-11 genes in the cardiac ventricular tissues of the mice. Notably, ECH treatment resulted in a significant improvement in the mRNA expression of SIRT1 and a reduction in the expression of IL-11 in cardiac tissue, with a *P*-value of less than 0.001 compared to the Ang II treatment group. To gain insights into systemic effects, we also measured the serum concentration of IL-11 using an ELISA assay. In the Ang II group, serum IL-11 levels were significantly higher at 140 pg/ml (*P*<0.001) compared to the control group, which had 52 pg/ml levels. However, in the ECH treatment group, the concentration of IL-11 was significantly lower, measuring 90 pg/ml, than in the Ang II-induced group (*P*<0.001)(Figure 4D).

In summary, our results indicate that ECH regulates the SIRT1/IL-11 pathway in the cardiac tissue of Ang II-infused mice, as evidenced by changes in protein and mRNA levels and systemic IL-11 concentration.

***Echinacoside inhibits Ang II-induced migration of rat cardiac fibroblasts***

We isolated primary cardiac fibroblasts from neonate SD rats’ ventricular myocytes and confirmed their identity through positive Vimentin staining (Figure 5A). These cardiac fibroblasts were pre-treated with a SIRT1 inhibitor, EX-527 (10 μM), and ECH (20 μM) for two hours, followed by a 48-hour incubation with Ang II (1 μM), after which cellular migration was evaluated using a Transwell assay (Figure 5B). Quantitative analysis revealed that ECH significantly reduced the number of migratory cells compared to Ang II-treated cardiac fibroblasts (*P*<0.001). Notably, the inclusion of the SIRT1 inhibitor EX-527 considerably attenuated the function of ECH and increased the number of migratory cells (*P*<0.001)(Figure 5C). To further explore these findings, we conducted RT-qPCR analysis to ascertain the mRNA expression levels of Collagen I (COL1A1) and Collagen III (COL3A1) in primary cardiac fibroblasts. Within the Ang II group pretreated with ECH, there was a notable decrease in the expression levels of both COL1A1 and COL3A1 compared to the Ang II group. However, in the ECH+EX-527 pretreatment group, we observed an elevated expression of COL1A1 and COL3A1 compared to the group treated with ECH alone (*P*<0.001) (Figure 5D). These results emphasize ECH’s role in reducing cardiac fibroblast migration and collagen deposition, and they underscore the influence of the SIRT1 pathway in this context.

***Echinacoside suppresses the Ang II-induced cardiac fibroblast differentiation***

We assessed the attenuating effects of ECH on Ang II-induced cardiac fibroblast differentiation. The differentiation of cardiac fibroblasts was determined by staining with α-SMA, a specific marker for myofibroblasts (Figure 6A). Quantitative analysis revealed that ECH significantly reduced the number of α-SMA positive cells in cardiac fibroblasts (*P*<0.001) compared to Ang II-induced cells. Interestingly, the number of α-SMA positive cells significantly increased in the ECH+EX-527 pretreatment group when compared to ECH pretreatment alone (*P*<0.001)(Figure 6B). In addition, RT-qPCR results demonstrated that ECH pretreatment led to a significant reduction in the mRNA expression of genes related to fibroblast differentiation and extracellular matrix (ECM) degradation, including α-SMA (ACTA2), Fibronectin, CTGF, and MMP-9 (*P*<0.001), as compared to Ang II-induced cells. In contrast, the ECH+EX-527 pretreatment group displayed a significant increase in the mRNA expression of these genes when compared to the ECH pretreatment group (*P*<0.001)(Figure 6C, D). These findings highlight ECH’s potential to suppress fibroblast differentiation and ECM degradation while underlining the influence of the SIRT1 pathway in modulating these processes.

***Echinacoside inhibits Ang II-induced intracellular ROS generation and oxidative stress***

We assessed intracellular ROS levels by staining with DHE (Figure 7A) and quantified ROS levels by determining the percentage of DHE-positive cells normalized to DAPI-stained cells. The analysis revealed that ECH markedly decreased the number of DHE-positive cells in primary fibroblasts from mice compared to those induced by Ang II (*P*<0.001). Intriguingly, the ECH+EX-527 pretreatment group displayed a significant increase in the number of DHE-positive cells compared with the ECH pretreatment alone (*P*<0.001)(Figure 7B). Furthermore, we measured oxidative stress-related markers, including MDA, SOD, and CAT, in cell lysates from cardiac fibroblasts. ECH pretreatment significantly improved the expression of MDA, SOD, and CAT in cardiac fibroblasts compared to those induced by Ang II (*P*<0.001)(Figure 7C, D, and E). Additionally, our RT-qPCR results indicated that the mRNA levels of two genes associated with oxidative stress, NOX2 and NOX4, were significantly reduced in ECH-pretreated cells compared to those induced by Ang II (*P*<0.001) (Figure 7F). However, ECH+EX-527 pretreatment resulted in a significant increase in the expression of these two genes than ECH pretreatment alone (*P*<0.001). These findings underscore ECH’s ability to mitigate ROS production and oxidative stress, with a clear influence of the SIRT1 pathway on these processes.

***Echinacoside enhances SIRT1 and regulates Wnt/β-Catenin and IL-11 pathway in Ang II-induced cardiac fibroblasts***

We conducted western blot analyses to assess SIRT1, IL-11, Wnt, and β-Catenin protein levels in Ang II-induced cardiac fibroblast cells, with representative protein bands in Figure 8A. ECH treatment significantly improved the SIRT1 protein expression and decreased the IL-11 expression, Wnt, and β-Catenin in Ang II-induced cardiac fibroblasts. Additionally, ECH+EX-527 therapy markedly reduced the SIRT1 expression and alleviated the IL-11 expression, Wnt, and β-Catenin in Ang II-induced cardiac fibroblasts. The protein quantification analysis revealed that SIRT1 expression was decreased to 0.25-fold, while IL-11, Wnt, and β-Catenin were increased to 3.10-fold, 2.8-fold, and 2.8-fold, respectively, compared to control cells in the Ang II-induced cells (*P*<0.001). In contrast, the protein quantification analysis of ECH-treated cells showed a 0.75-fold alleviation in SIRT1 expression and a 1.50-fold, 1.25-fold, and 1.25-fold reduction in the expression of IL-11, Wnt, and β-Catenin, respectively, compared to the Ang II-induced cells (*P*<0.001)(Figure 8B, C, D, E). The above results emphasize the role of ECH in modulating the critical protein expression involved in CF and suggest the involvement of the SIRT1/IL-11 pathway in this process.

## Discussion

Fibrosis represents a critical pathological feature in a range of advanced cardiovascular diseases (1). This research aimed to explore the molecular mechanisms contributing to the attenuating effects of ECH against Ang II-infused CF. In our *in vivo* experiments, ECH effectively mitigated cardiac remodeling and dysfunction, reduced the collagen volume fraction, decreased the expression of fibrosis-associated markers (collagen I and collagen III), lowered the levels of myocardial enzymes (ANP, BNP, CK-MB, cTnl, and cTnT), and suppressed cardiac cell apoptosis (down-regulating BAX and up-regulating BCL2). ECH also exerted a significant regulatory influence on both mRNA and protein levels of SIRT1 and IL-11. In our *in vitro* studies, ECH pretreatment exhibited a protective effect by reversing fibrosis in cardiac fibroblasts, impacting the same processes mentioned earlier. Our findings strongly indicate the involvement of SIRT1 and IL-11 in ECH’s protective effects against CF, which was corroborated through the use of the SIRT1 inhibitor, EX-527.

Reducing collagen deposition is crucial, as it can ameliorate diastolic stiffness and improve relaxation, thus mitigating left ventricular dysfunction (39,40). Collagens I and III are the principal components of the cardiac ECM, and their synthesis is crucial for effective wound healing. Additionally, ECM regulatory proteins and cytokines play pivotal roles in ECM homeostasis, and the deletion of MMP-9 has been demonstrated to attenuate collagen deposition (41). Our study revealed that Ang II-induced mice displayed improved mRNA levels of collagen I and collagen III in left ventricular heart tissue, which was effectively attenuated by ECH treatment. Cardiac injury markers such as myocardial cardiac enzymes (CK-MB, cTnT, ANP, and BNP) are widely employed to assess various cardiac dysfunctions due to their high sensitivity and specificity (42). Prior studies have reported elevated serum levels of CK-MB, cTnT, ANP, and BNP in various heart diseases (43).

SIRT1 and IL-11 are recognized as pivotal drivers in fibrosis development (20,21,27,30,44,45). IL-11 expression is correlated with fibroblast activation, and the promotion of fibroblast-to-myofibroblast transition is facilitated by the WNT/β-catenin signaling pathway through the enhancement of IL-11 production, as documented in previous studies (45-47). Nevertheless, whether SIRT1 and IL-11 play a role in ECH-mediated protection against Ang II-induced fibrosis remained unclear. Our research demonstrated that SIRT1 expression decreased after Ang II-infused CF, both *in vivo* and *in vitro*, and that ECH treatment mitigated SIRT1 down-regulation and IL-11 up-regulation. To substantiate the roles of SIRT1 and IL-11 in the anti-fibrotic process, cardiac fibroblasts were pre-treated with a SIRT1 inhibitor (EX-527) before exposure to ECH and Ang II treatments. Our findings unveiled that inhibiting SIRT1 heightened the expression of IL-11, Wnt, and β-catenin and collagen deposition while suppressing the expression and functionality of MMPs (MMP-9). Additionally, it was observed that the inhibition of SIRT1 compromised the anti-apoptotic and anti-migratory properties of ECH after Ang II stimulation. Additionally, intracellular ROS, oxidative stress markers (MDA, SOD, and CAT), and related genes (NOX2 and NOX4) were up-regulated in Ang II-treated cardiac fibroblasts after ECH+EX-527 treatment. These outcomes collectively suggest that the SIRT1/IL-11 pathway is a crucial player in the anti-fibrotic impact of ECH in the context of cardiac fibrosis.

Nonetheless, there are limitations to our study, and additional investigation is required to comprehensively clarify the fundamental mechanisms accountable for the beneficial impacts of SIRT1 and IL-11 in the context of CF. In this present research, the dose-dependent effect of ECH on Ang II-infused CF was not elucidated. Future investigations should consider various ECH doses to improve a more inclusive understanding of the dose-dependent nature of ECH’s effectiveness in mitigating CF. Further in-depth research is warranted for a more precise evaluation of Ang II-infused CF and the attenuation of fibrosis by ECH.

## Conclusion

ECH effectively reduced CF by controlling collagen deposition and ECM degradation, as well as the apoptosis and movement of cardiac fibroblasts. These protective effects of ECH were primarily due to the activation of SIRT1 and the decrease of IL-11. Our study provides new insights into the mechanism behind ECH’s anti-fibrotic effects through the SIRT1/IL-11 pathway. Targeting the SIRT1/IL-11 pathway may alleviate CF-related dysfunction. Therefore, ECH could be deemed a promising therapeutic intervention for enhancing the management of CF in individuals.

## Declaration

We have not used any AI tools or technologies to prepare this manuscript.
